# Do rats see like we see?

**DOI:** 10.7554/eLife.26401

**Published:** 2017-04-12

**Authors:** Nicole C Rust

**Affiliations:** Department of Psychology, University of Pennsylvania, Philadelphia, United Statesnrust@sas.upenn.edu

**Keywords:** object recognition, visual cortex, invariance, transformation tolerance, neuronal coding, information theory, Rat

## Abstract

Like primates, the rat brain areas thought to be involved in visual object recognition are arranged in a hierarchy.

**Related research article** Tafazoli S, Safaai H, De Franceschi G, Rosselli FB, Vanzella W, Riggi M, Buffolo F, Panzeri S, Zoccolan D. 2017. Emergence of transformation-tolerant representations of visual objects in rat lateral extrastriate cortex. *eLife* **6**:e22794. doi: 10.7554/eLife.22794

In our eyes, cells called photoreceptors convert the world around us into a pixel-like representation. Our brains must then reorganize this into a representation that reflects the identities of the objects we are looking at. The same object can be represented by very different pixel patterns, depending on its distance from us, the viewing angle and the lighting conditions. Conversely, different objects can be represented by pixel patterns that are similar. This is what makes object recognition a tremendously challenging problem for our brains to solve, and we do not fully understand how our brains manage to recognize objects.

Nonhuman primates (such as rhesus monkeys) are routinely used to study object recognition because their brains are similar to ours in many ways. However, there are advantages to working with mice and rats, including access to an array of modern biotechnological tools that have been optimized for these species. These tools include sophisticated ways to measure neural activity (Svoboda and Yasuda, 2006), to manipulate neural activity (Fenno et al., 2011), and to map how neurons are connected together within and between brain areas (Oh et al., 2014).

Skepticism that rodents could be used to gain insight into object recognition has largely been targeted at the ways in which rodent visual systems deviate from our own. For example, the retinae of mice and rats are specialized for seeing in the dark, and they lack a region called the fovea that allows humans to see objects in great detail at the center of the gaze. The visual cortex is also organized differently in primates and rodents with regard to how neurons with similar preferences for visual stimuli are clustered together within each brain area, and a much smaller fraction of the rodent cortex is devoted to visual processing. In light of all of these differences, can we really learn much about how our brains recognize objects by studying how rodents see?

In an earlier study, Davide Zoccolan and colleagues presented behavioral evidence that rats are capable of identifying objects under variations in viewing conditions (Zoccolan et al., 2009). Now, in eLife, Zoccolan and co-workers at SISSA in Trieste, the Istituto Italiano di Tecnologia and Harvard Medical School – including Sina Tafazoli and Houman Safaai as joint first authors – present evidence that this behavior is supported by four visual areas of the brain that are arranged in a functional hierarchy (Tafazoli et al., 2017). This is analogous to how object processing happens in the primate brain (DiCarlo et al., 2012).

Researchers had previously relied on anatomical evidence to argue that visual brain areas in rats are organized in a hierarchical fashion (Coogan and Burkhalter, 1993). Tafazoli et al. recorded the activity of four of these areas – termed V1, LM, LI and LL – in response to different objects as they systematically changed a number of variables (such as the position, size and luminance of each object). With this data, they quantified how much information each brain area reflected about the identity of the object, as well as how that information was formatted.

A key insight came from analyzing the degree to which changes in the neural responses to different objects could be attributed to differences in object luminance as opposed to object shape. Compared to the other brain areas, the firing rate of the neurons in V1 (the first brain area in the hierarchy) depended more strongly on the amount of luminance within the region of the visual field that each neuron was sensitive to. Moving through the hierarchy, an increasingly large proportion of the responses of the neurons reflected information about the shape of the object. At the same time, there was a systematic increase in the degree to which information about object identity was formatted in a manner that would make it easy for higher brain areas to access this information (DiCarlo and Cox, 2007).

In the face of considerable evidence that object processing in rats and primates is different, Tafazoli et al. have uncovered a compelling similarity. By design, their study has strong parallels with the studies that established a hierarchy for object processing in the primate brain, and their results suggest that rats and primates may perform object recognition in broadly similar ways. Future work will be required to determine the degree to which the nuts-and-bolts of object processing are in fact the same between the species.

## References

[bib1] Coogan TA, Burkhalter A (1993). Hierarchical organization of areas in rat visual cortex. Journal of Neuroscience.

[bib2] DiCarlo JJ, Cox DD (2007). Untangling invariant object recognition. Trends in Cognitive Sciences.

[bib3] DiCarlo JJ, Zoccolan D, Rust NC (2012). How does the brain solve visual object recognition?. Neuron.

[bib4] Fenno L, Yizhar O, Deisseroth K (2011). The development and application of optogenetics. Annual Review of Neuroscience.

[bib5] Oh SW, Harris JA, Ng L, Winslow B, Cain N, Mihalas S, Wang Q, Lau C, Kuan L, Henry AM, Mortrud MT, Ouellette B, Nguyen TN, Sorensen SA, Slaughterbeck CR, Wakeman W, Li Y, Feng D, Ho A, Nicholas E, Hirokawa KE, Bohn P, Joines KM, Peng H, Hawrylycz MJ, Phillips JW, Hohmann JG, Wohnoutka P, Gerfen CR, Koch C, Bernard A, Dang C, Jones AR, Zeng H (2014). A mesoscale connectome of the mouse brain. Nature.

[bib6] Svoboda K, Yasuda R (2006). Principles of two-photon excitation microscopy and its applications to neuroscience. Neuron.

[bib7] Tafazoli S, Safaai H, De Franceschi G, Rosselli FB, Vanzella W, Riggi M, Buffolo F, Panzeri S, Zoccolan D (2017). Emergence of transformation-tolerant representations of visual objects in rat lateral extrastriate cortex. eLife.

[bib8] Zoccolan D, Oertelt N, DiCarlo JJ, Cox DD (2009). A rodent model for the study of invariant visual object recognition. PNAS.

